# Supplementary material for: Novel Mesogenic Vinyl Ketone Monomers and Their Based Polymers
Source: Polymers (Basel). 2022 Dec 20;15(1):5. doi: 10.3390/polym15010005 (PMC9823505; doi:10.3390/polym15010005)
Supplement: Supplementary file 1 [file polymers-15-00005-s001.zip › polymers-2092001-supplementary.pdf]

Novel mesogenic vinyl ketone monomers and their based polymers

Derikov Y.I. [1], **Belousov D.R.**[1,2], **Finko A.V.** [1,3], **Shandryuk G.A.**[1], **Kuz'menok N.M.**[4], **Mikhalyonok S.G.**[4], **Bezborodov V.S.**[4] **and Chernikova E.V.**[1,3],**Talroze R.V.**[1*]

[1] A.V. Topchiev Institute of Petrochemical Synthesis Russian Academy of Sciences, Moscow119991, Russia
[2] D. Mendeleev University of Chemical Technology of Russia, Moscow, 125047 Russia
[3] M.V. Lomonosov Moscow State University, Faculty of Chemistry, Moscow, 119991 Russia
[4] Belarusian State Technological University. Department of Chemistry, Minsk, 220006 Belarus
* Correspondence: rtalroze@ips.ac.ru

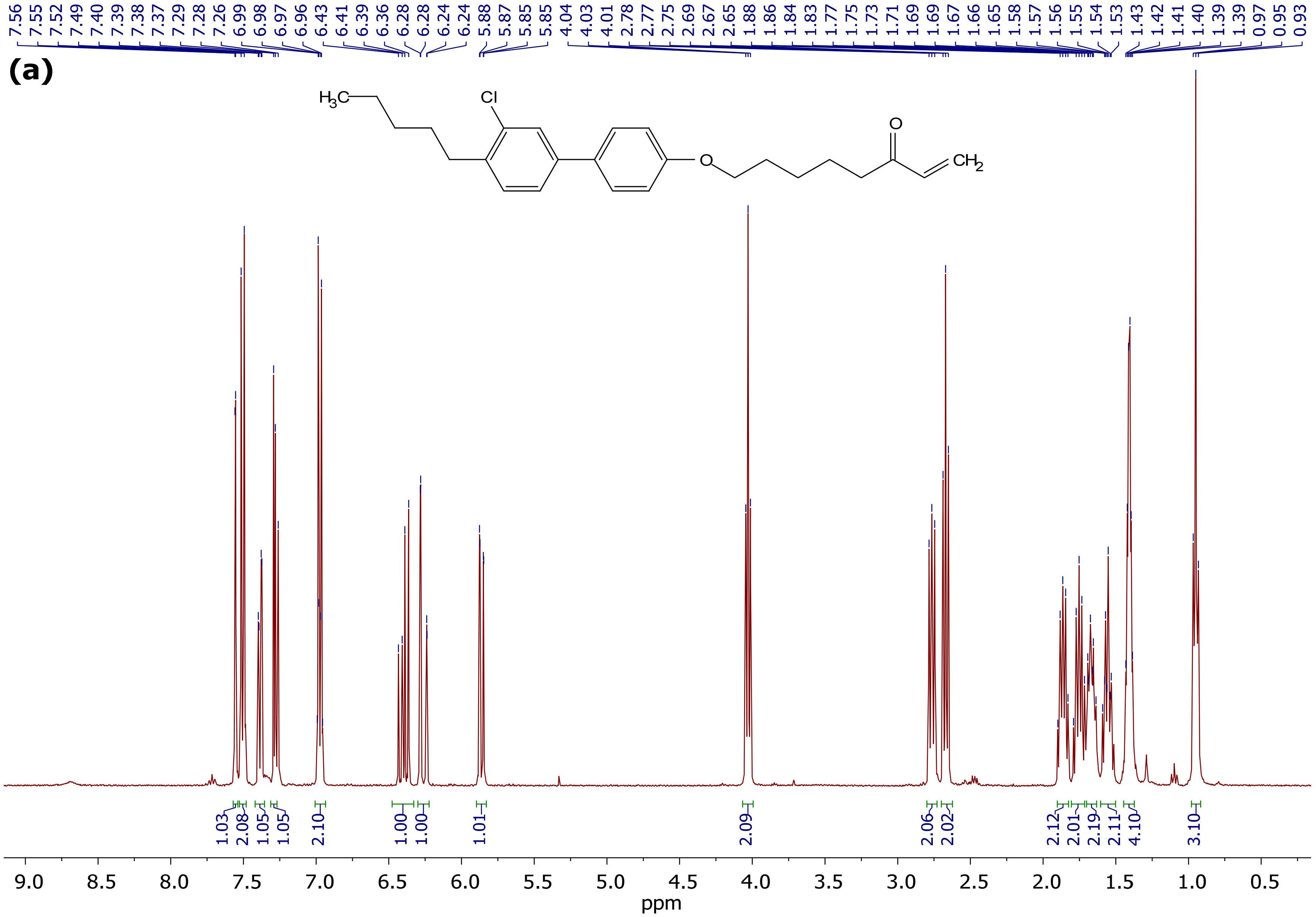

(a)
7.56 7.55 7.52 7.49 7.40 7.39 7.38 7.37 7.29 7.28 7.26 6.99 6.98 6.97 6.96 6.43 6.41 6.39 6.36 6.28 6.28 6.24 6.24 5.88 5.87 5.85 5.85 4.04 4.03 4.01 2.78 2.77 2.75 2.69 2.67 2.65 1.88 1.86 1.84 1.83 1.77 1.75 1.73 1.71 1.69 1.69 1.67 1.66 1.65 1.58 1.57 1.56 1.55 1.54 1.53 1.43 1.42 1.41 1.40 1.39 1.39 0.97 0.95 0.93
H3C
Cl
O
O
CH2
1.03 2.08 1.05 1.05 2.10 1.00 1.00 1.01 2.09 2.06 2.02 2.12 2.01 2.19 2.11 4.10 3.10
9.0 8.5 8.0 7.5 7.0 6.5 6.0 5.5 5.0 4.5 4.0 3.5 3.0 2.5 2.0 1.5 1.0 0.5
ppm

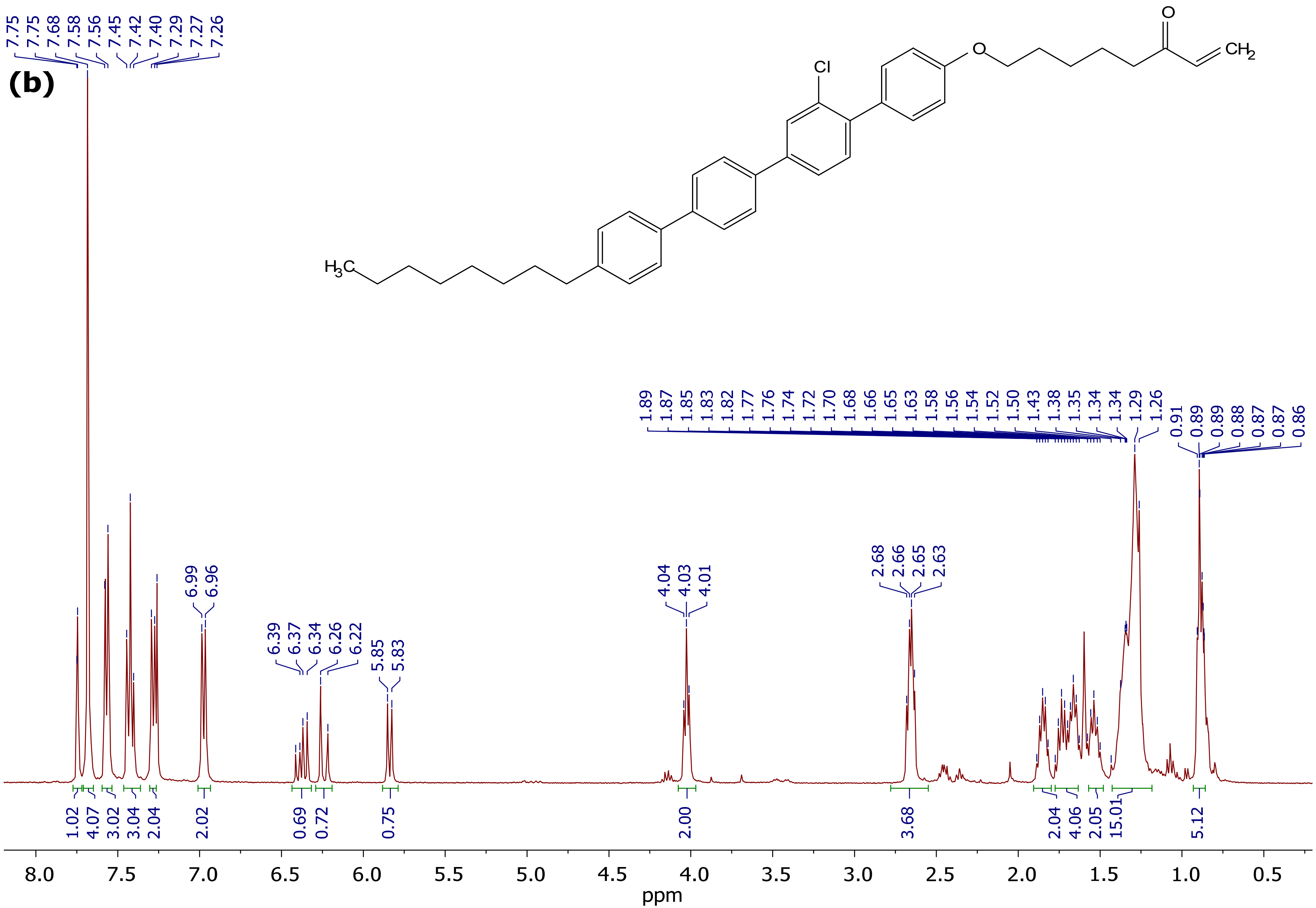

Figure S1. The ^{1}H spectra of BVK (a) and QVK (b) in $CDCl_3$.

(a)

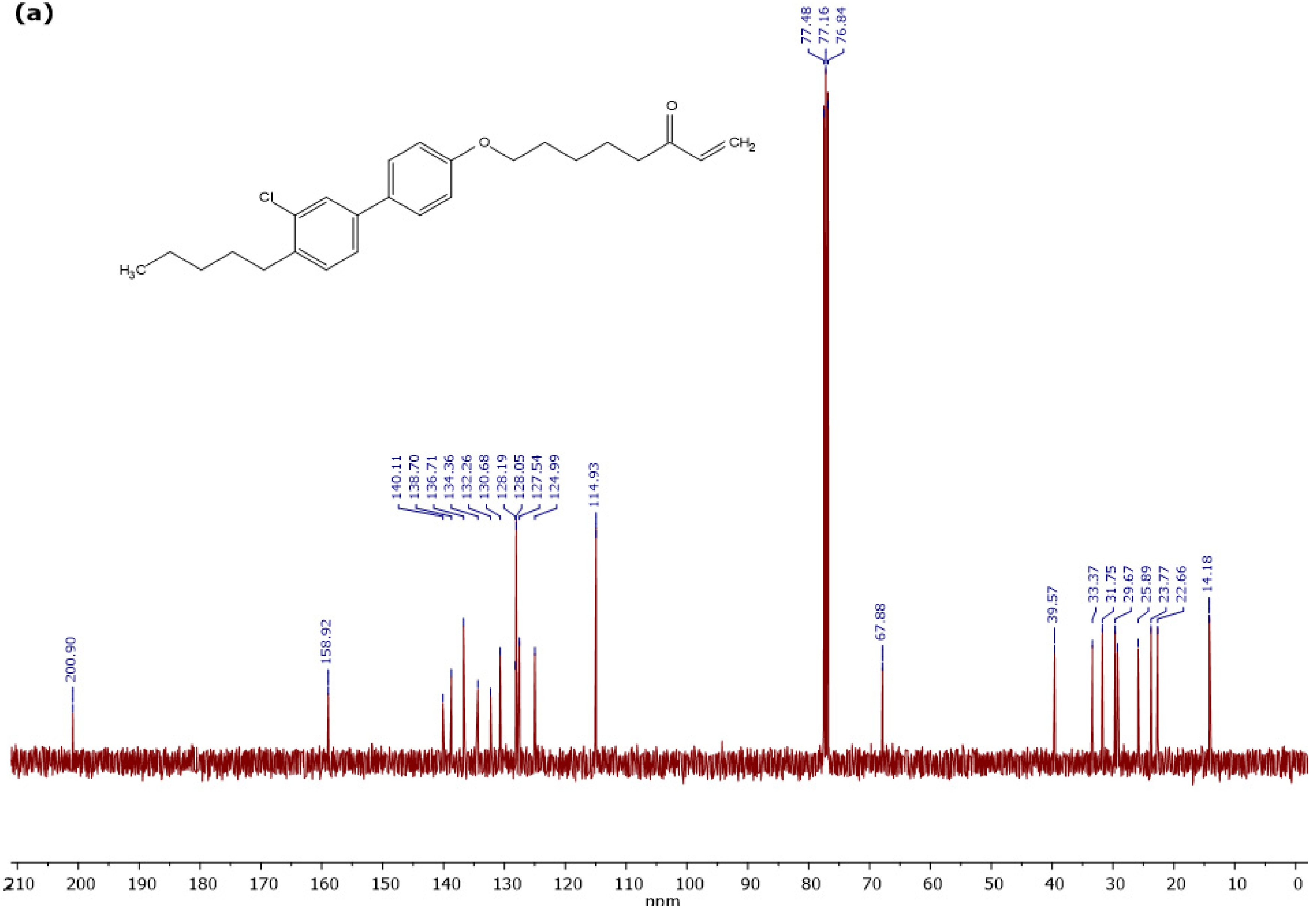
O
CH_2
Cl
H_3C
77.48
77.16
76.84
140.11
138.70
136.71
134.36
132.26
130.68
128.19
128.05
127.54
124.99
114.93
200.90
158.92
67.88
39.57
33.37
31.75
29.67
25.89
23.77
22.66
14.18
210 200 190 180 170 160 150 140 130 120 110 100 90 80 70 60 50 40 30 20 10 0
ppm

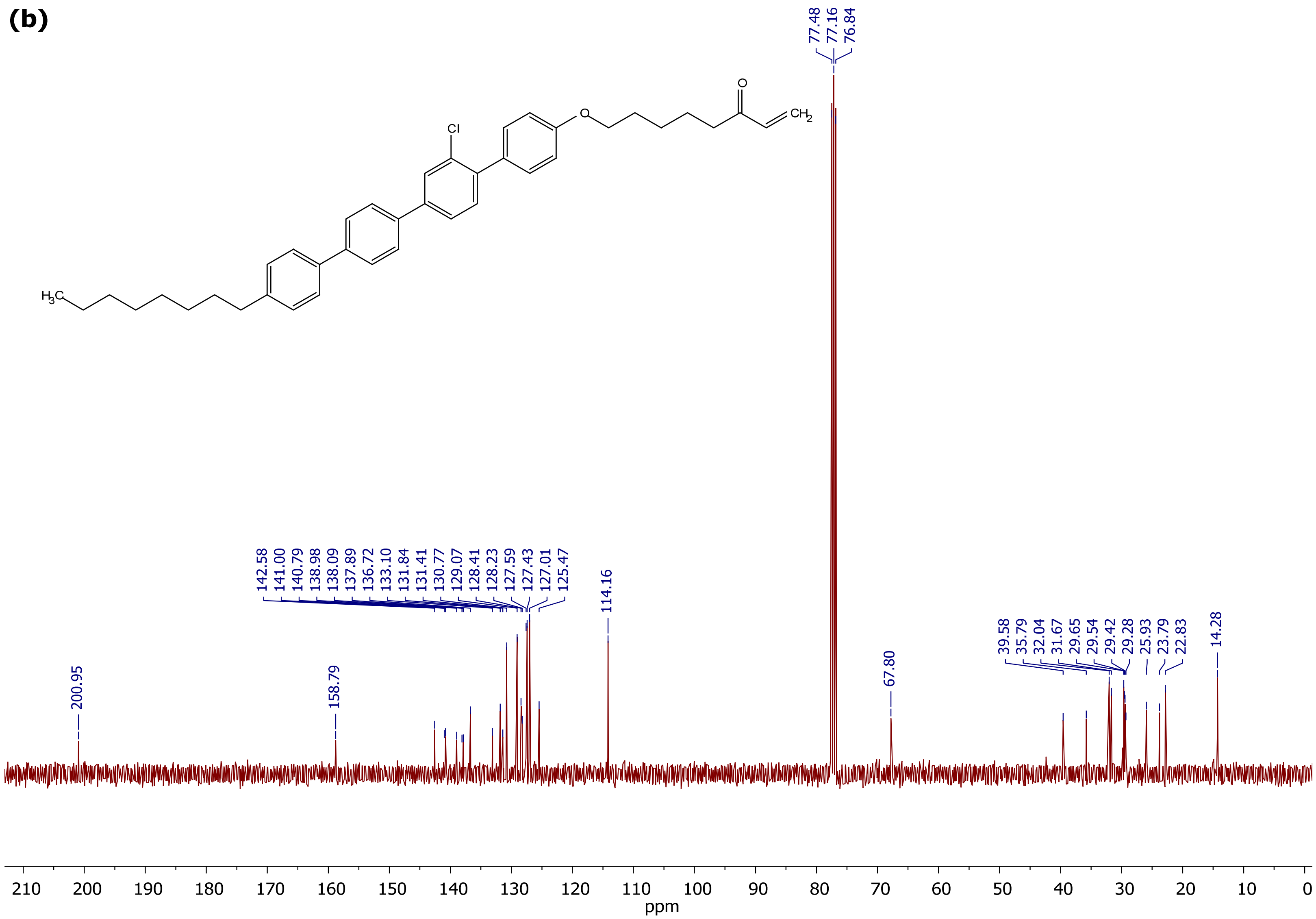

Figure S2. The ^{13}C spectra of BVK (a) and QVK (b) monomers in $CDCl_3$.

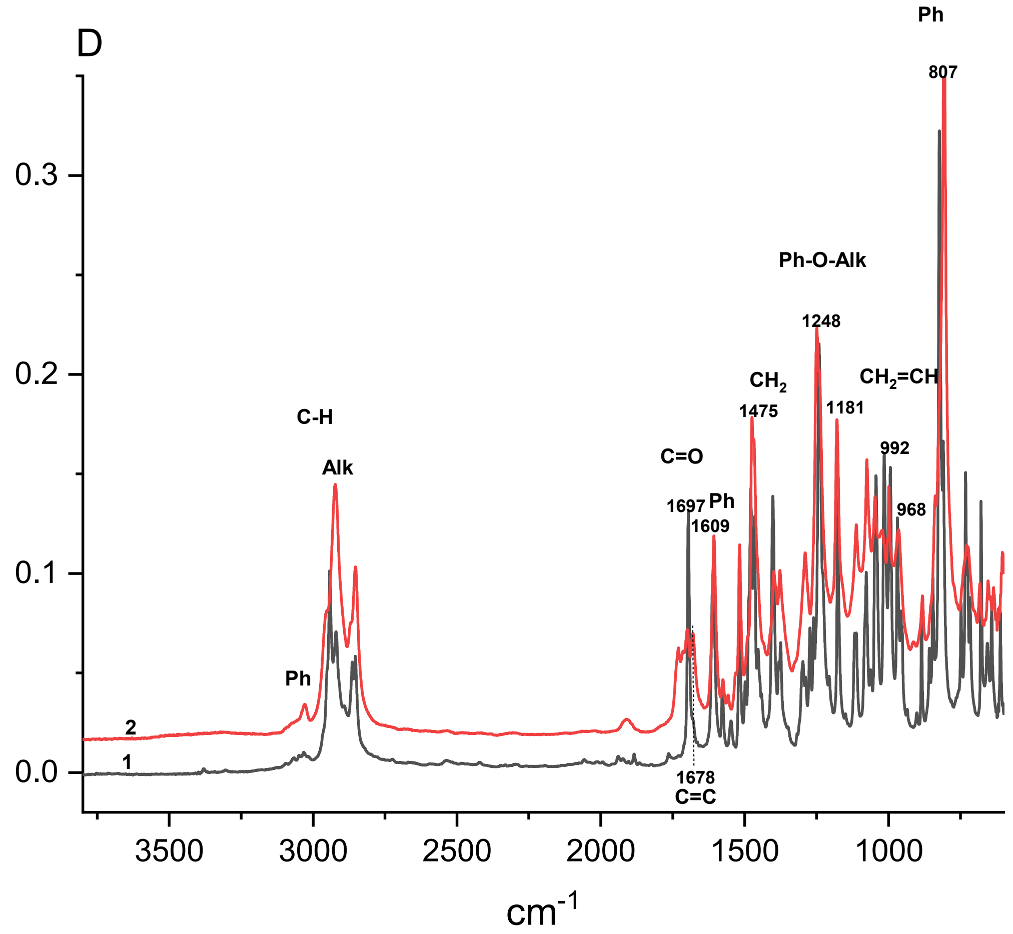

Figure S3. IR spectra of BVK(1) and QVK (2)

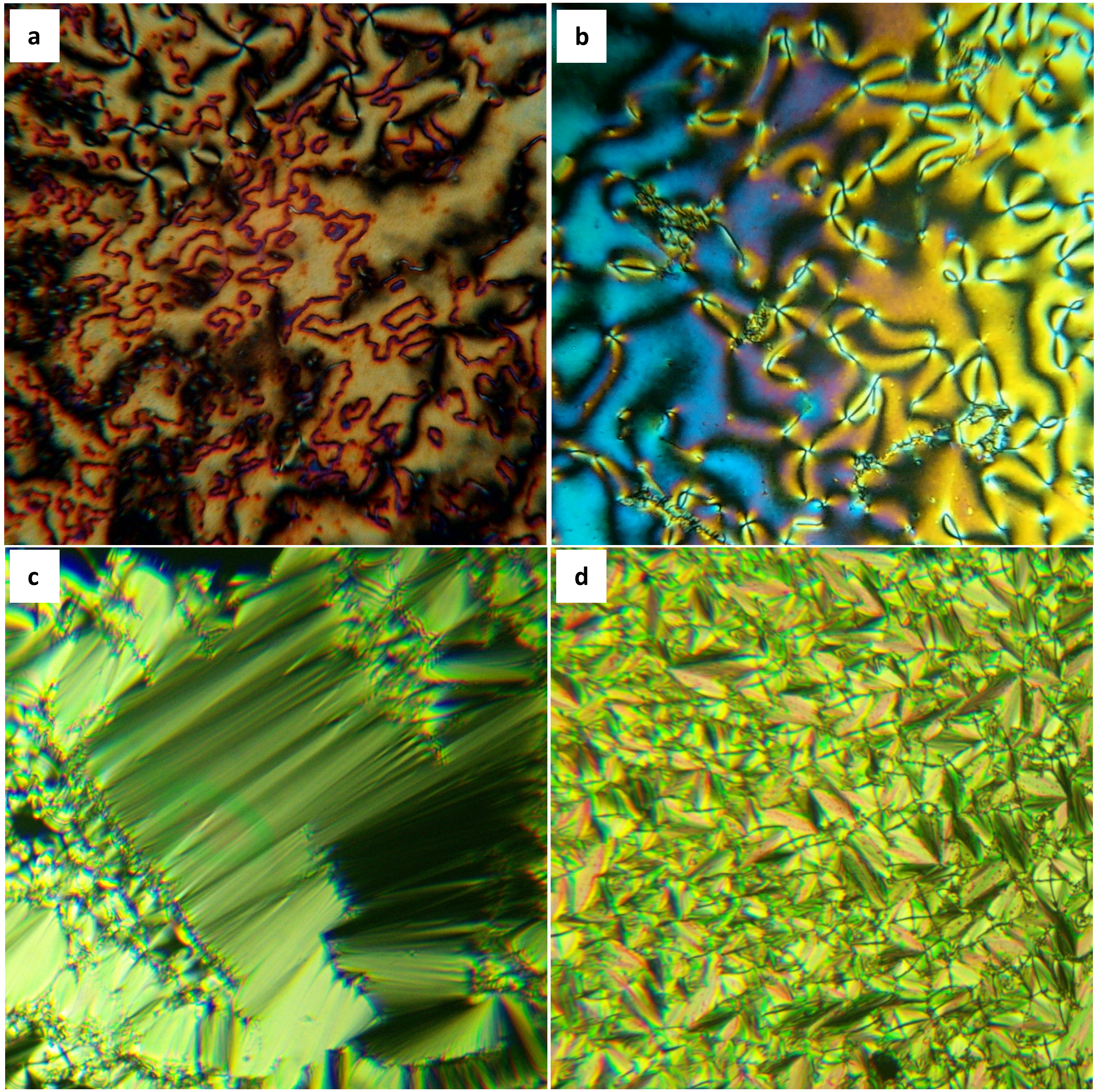

Figure S4. Thread-like/schlieren mixed texture of TVK (a) and schlieren texture of poly(TVK) (b); fan-shaped texture of QVK (c) and focal conic texture of poly(QVK)
